# Supplementary material for: Mycotoxins in Tea ((Camellia sinensis (L.) Kuntze)): Contamination and Dietary Exposure Profiling in the Chinese Population
Source: Toxins (Basel). 2022 Jul 1;14(7):452. doi: 10.3390/toxins14070452 (PMC9318285; doi:10.3390/toxins14070452)
Supplement: Supplementary file 1 [file toxins-14-00452-s001.zip › toxins-1765816-supplementary.pdf]

Supplementary Materials: Mycotoxins in Tea ((*Camellia sinensis* (L.) Kuntze)): Contamination and Dietary Exposure Profiling in the Chinese Population

Haiyan Zhou, Zheng Yan, Aibo Wu, and Na Liu*

Table S1. Average dietary consumption of tea by persons in different area and their average body weight.

Area	N (person)	Bw (kg)	CA ($g \cdot day^{-1}$)	Tea Categories
Mongolia	99	67.60	3.70	**Dark tea**, green tea, black tea, oolong tea, and others. (Ye et al.)
Kunming	109	60.00	3.47/1.50	**Pu-erh fermented tea/raw tea**, green tea, black tea, oolong tea, and others. (Cao et al.)
Pu'er	110	62.80	4.39/4.64	
Harbin	57555		4.20	
Qingdao	35509		4.10	
Suzhou	53260		4.10	
Liuzhou	50173	59.69	4.10	**Green tea**, black tea, oolong tea, and others. (Chen et al. and Guan et al.)
Haikou	29689		4.20	
Henan	63357		4.10	
Gansu	50041		4.10	
Sichuan	55687		4.10	
Zhejiang	57704		4.00	
Hunan	59916		4.10	

Table S2. Concentration range and mean of mycotoxins calculated with upper bound in all tea samples ($\mu g \cdot kg^{-1}$).

Mycotoxin	MRLs	Green Tea (81)				Oolong Tea (64)				Black Tea (104)				Dark Tea (103)			
		PS	Max	Mean	SD	PS	Max	Mean	SD	PS	Max	Mean	SD	PS	Max	Mean	SD
AFB_1	5[A]	0	0.13	0.03	0.02	0	0.03	0.03	-	0	0.14	0.03	0.01	0	1.11	0.06	0.14
AFB_2	NF	-	0.29	0.02	0.04	-	0.61	0.03	0.08	-	0.41	0.02	0.04	-	0.32	0.06	0.07
AFG_1	NF	-	0.10	0.03	0.01	-	2.78	0.28	0.47	-	15.36	0.58	2.24	-	4.34	0.53	0.85
AFG_2	NF	-	6.75	1.21	1.32	-	13.36	5.81	3.69	-	47.08	5.33	4.89	-	38.10	9.93	6.51
AFs	20[A]	0	6.82	1.29	1.31	0	14.31	6.14	3.75	2	47.15	5.96	5.53	7	39.57	10.58	6.71
ZEN	75[B]	0	1.75	0.17	0.20	0	0.79	0.18	0.14	0	5.74	0.67	1.03	0	8.41	2.10	2.57
α-ZEL	NF	-	24.71	17.83	4.18	-	48.03	23.38	12.21	-	116.22	24.05	21.16	-	99.40	19.49	19.46
β-ZEL	NF	-	93.55	30.84	8.84	-	125.70	75.32	19.92	-	873.37	96.35	90.71	-	137.39	57.34	20.08
α-ZAL	NF	-	0.75	0.75	-	-	12.87	0.94	1.52	-	0.75	0.75	-	-	14.08	1.11	1.56
β-ZAL	NF	-	0.75	0.75	-	-	0.75	0.75	-	-	0.75	0.75	-	-	0.75	0.75	-
DON	750[B]	0	120.07	15.88[c]	21.78	6	1748.09	183.97[a]	364.91	3	985.47	84.85[b]	196.19	6	1131.13	122.16[a]	260.72
15-Ac DON	NF	-	38.34	16.34	4.59	-	15	15	-	-	748.13	56.49	121.69	-	736.16	84.70	137.47
3-Ac DON	NF	-	7.5	7.08	1.39	-	8.03	7.42	0.52	-	36.56	7.78	2.85	-	12.77	7.61	0.62
OTA	5[B]	0	0.56	0.09[b]	0.08	0	1.06	0.33[b]	0.28	0	4.90	1.33[b]	0.96	4	11354.64	129.76[a]	1128.93
NEO	200[C]	0	7.27	0.99	1.08	0	12.07	1.19	1.90	0	45.95	1.28	4.45	0	35.68	4.98	8.38
T-2	200[B]	0	1.46	1.45	0.18	0	1.52	1.50	0.002	0	16.01	1.64	1.42	0	36.88	2.57	5.52
CIT	200[D]	0	17.46	0.93	2.84	0	91.90	8.97	18.31	0	93.27	6.97	16.51	1	203.76	20.87	35.27

NF: Not Found; MRLs: relevant-Maximum Regulation Limits; PS: positive samples exceeding for MRLs (number).

[A] Limits for AFB_1 and AFs in raw tea materials (TRCU, 2011)

[B] MRLs of contaminants in foods (EC Regulation No 1881/2006).

[C] MRLs with respect to T-2 MRLs (EC Regulation No 1881/2006).

[D] MRLs with respect to the evaluation of citrinin occurrence in Chinese Liupao tea by Li et al.

Table S3. The lower bound probabilistic estimation of six groups mycotoxins exposure on four types of tea consumption (μg·kg^{-1}·day^{-1}).

**Mycotoxins**	**DONs**	**ZENs**	**NEO**	**OTA**	**T-2**	**CIT**
PMTDI	1	0.25	0.1	0.0143	0.1	0.2
Mean						
Green Tea	9.09 E-04	3.41 E-03	3.76 E-05	2.95 E-06	5.64 E-06	5.94 E-05
Oolong Tea	1.11 E-02	6.12 E-03	3.50 E-04	1.28 E-03	1.46 E-05	2.76 E-03
Black Tea	7.67 E-03	7.51 E-03	3.61 E-05	8.27 E-05	9.57 E-06	4.30 E-04
Dark Tea	1.06 E-02	4.44 E-03	2.48 E-04	8.58 E-03	6.29 E-05	1.15 E-03
P50						
Green Tea	6.27 E-04	3.34 E-03	2.59 E-05	2.03 E-06	3.89 E-06	4.09 E-05
Oolong Tea	7.62 E-03	5.79 E-03	2.41 E-04	8.80 E-04	1.01 E-05	1.90 E-03
Black Tea	5.30 E-03	5.80 E-03	2.49 E-05	6.97 E-05	6.60 E-06	2.97 E-04
Dark Tea	7.31 E-03	4.00 E-03	1.71 E-04	1.57 E-07	4.34 E-05	7.94 E-04
P95						
Green Tea	2.75 E-03	4.78 E-03	1.14 E-04	8.90 E-06	1.70 E-05	1.79 E-04
Oolong Tea	3.35 E-02	9.82 E-03	1.06 E-03	3.87 E-03	4.43 E-05	8.34 E-03
Black Tea	2.31 E-02	1.82 E-02	1.09 E-04	2.02 E-04	2.88 E-05	1.30 E-03
Dark Tea	3.19 E-02	8.73 E-03	7.47 E-04	2.13 E-05	1.89 E-04	3.47 E-03

Table S4. The middle bound probabilistic estimation of six groups mycotoxins exposure on four types of tea consumption (μg·kg^{-1}·day^{-1}).

Mycotoxins	DONs	ZENs	NEO	OTA	T-2	CIT
PMTDI	1	0.25	0.1	0.0143	0.1	0.2
Mean						
Green Tea	1.39 E-03	3.46 E-03	3.79 E-05	4.34 E-06	5.16 E-05	5.14 E-06
Oolong Tea	7.92 E-02	6.16 E-03	3.19 E-05	1.94 E-05	4.76 E-05	5.52 E-04
Black Tea	2.64 E-03	7.58 E-03	2.76 E-05	8.29 E-05	4.85 E-05	8.08 E-03
Dark Tea	1.39 E-02	2.67 E-02	2.63 E-04	1.72 E-04	4.94 E-05	1.15 E-03
P50						
Green Tea	1.19 E-03	3.39 E-03	3.03 E-05	3.55 E-06	5.16 E-05	3.14 E-06
Oolong Tea	1.37 E-03	5.83 E-03	2.82 E-05	1.41 E-05	4.74 E-05	3.81 E-04
Black Tea	1.38 E-03	5.83 E-03	2.61 E-05	6.83 E-05	4.80 E-05	6.02 E-06
Dark Tea	1.52 E-03	3.96 E-03	3.87 E-05	1.56 E-05	4.65 E-05	7.95 E-04
P95						
Green Tea	2.30 E-03	4.83 E-03	8.23 E-05	9.94 E-06	5.62 E-05	1.26 E-05
Oolong Tea	2.04 E-02	9.84 E-03	5.17 E-05	5.39 E-05	4.86 E-05	1.67 E-03
Black Tea	6.12 E-03	1.83 E-02	3.67 E-05	2.05 E-04	5.14 E-05	2.88 E-04
Dark Tea	1.52 E-02	9.30 E-03	1.07 E-03	5.66 E-04	6.60 E-05	3.47 E-03

Table S5. The upper bound probabilistic estimation of carcinogenic risk (R) of AFG_2 through dark tea consumption in different regions.

**Groups**	**Ulan Bator of Mongolia**			**Kunming**			**Pu'er**		
	**Mean**	**P50**	**P95**	**Mean**	**P50**	**P95**	**Mean**	**P50**	**P95**
Male	6.17 E-06	5.65 E-06	1.35 E-05	2.80 E-06	2.56 E-06	5.06 E-06	7.43 E-06	6.79 E-06	1.34 E-05
Female	3.33 E-06	3.05 E-06	7.27 E-06	5.03 E-06	4.60 E-06	9.09 E-06	4.02 E-06	3.68 E-06	7.26 E-06
30-39	3.46 E-06	3.16 E-06	7.54 E-06	4.84 E-06	4.43 E-06	8.74 E-06	6.2 7 E-06	5.73 E-06	1.13 E-05
40-49	3.24 E-06	2.97 E-06	7.08 E-06	2.78 E-06	2.54 E-06	5.02 E-06	3.65 E-06	3.34 E-06	6.60 E-06
≥50	6.97 E-06	6.38 E-06	1.52 E-05	4.82 E-06	4.41 E-06	8.71 E-06	1.01 E-05	9.21 E-06	1.82 E-05

The bold fonts represent R >1.0 E-05.

Table S6. The deterministic estimation of mycotoxins exposure on green tea powder consumption ($\mu g \cdot kg^{-1} \cdot day^{-1}$).

Mycotoxins	Lower Bound	Middle Bound	Upper Bound
DONs	2.08E-04	4.10E-04	6.11E-04
ZENs	7.58E-04	7.70E-04	7.83E-04
NEO	8.60E-06	1.20E-05	1.54E-05
OTA	6.74E-07	1.02E-06	1.37E-06
T-2	1.29E-06	1.19E-05	2.26E-05
CIT	1.36E-05	1.40E-05	1.44E-05
AFs	1.90E-05	1.96E-05	2.01E-05

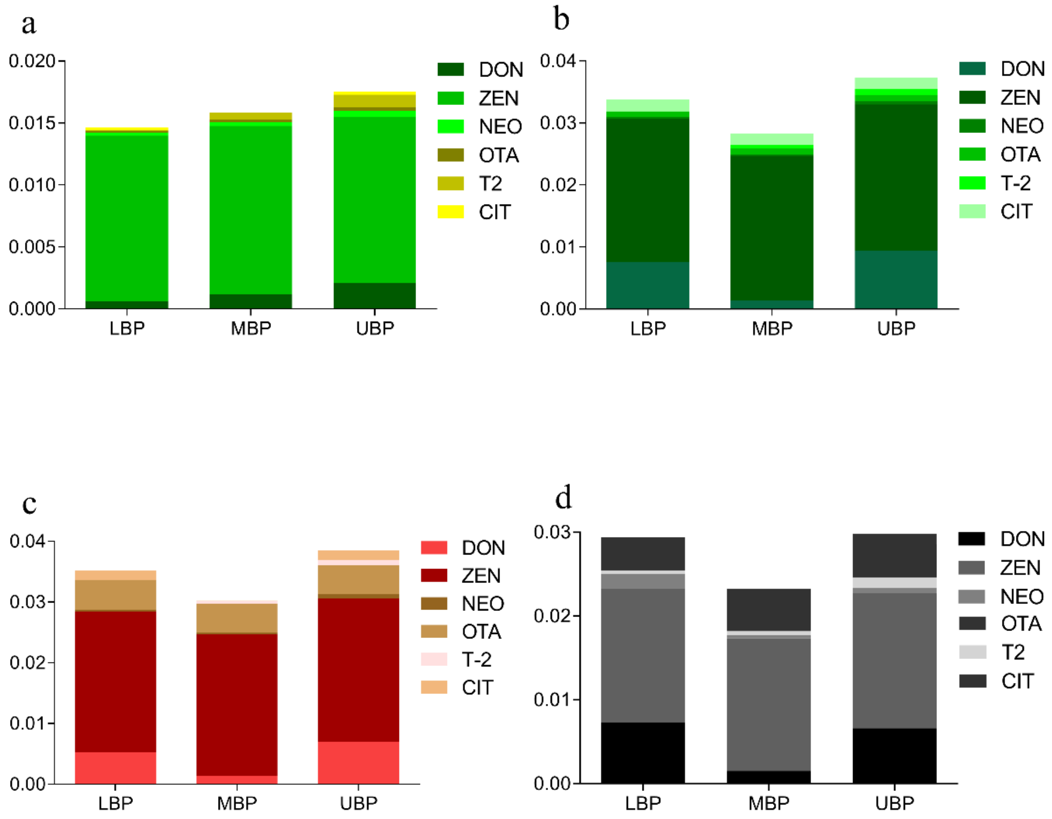

Figure S1. The (HI) values of six non-carcinogenic mycotoxins from tea consumption are the sum of MOS values (P50) from probabilistic estimation with three bounds: a) green tea, b) oolong tea, c) black tea, d) dark tea.
